# Maternal–Fetal Transfer of Domoic Acid in Rats at Two Gestational Time Points

**DOI:** 10.1289/ehp.10446

**Published:** 2007-09-19

**Authors:** Jennifer M. Maucher, John S. Ramsdell

**Affiliations:** Marine Biotoxins Program, Center for Coastal Environmental Health and Biomolecular Research, National Oceanic and Atmospheric Administration—National Ocean Service, Charleston, South Carolina, USA

**Keywords:** California sea lion, domoic acid, prenatal exposure, rats

## Abstract

**Background and objectives:**

Prenatal exposure to asymptomatic doses of domoic acid (DA) causes learning and memory deficits later in life; therefore, we sought to measure distribution of DA in maternal plasma and brain, prenatal brain, and amniotic fluid 1 hr after exposure, a time frame that normally encompasses acute seizure behavior.

**Methods:**

Pregnant rats were given a single intravenous dose of DA (0.6 or 1.6 mg/kg body weight) at either gestational day (GD) 13 or GD20, which correspond to the beginning of rat embryo neurogenesis and the last day of gestation, respectively. Using a direct ELISA, dose-dependent levels of DA were detected in each sample matrix tested.

**Results:**

An average of 6.6 and 14 ng DA/g brain tissue was found in GD13 and GD20 prenatal rats, respectively. Brain concentrations of DA in the GD13 prenates were identical to amniotic fluid levels, consistent with no restriction for DA to enter the GD13 prenatal brain. At GD20 the prenatal brain contained half the concentration of DA in the amniotic fluid, and was approximately half that found in the brain of the dams. After 1 hr, fetal brain and amniotic fluid contained between 1 and 5% of DA found in the maternal circulation. The amniotic fluid levels of DA in this study were also within the same range measured in stranded California sea lions that showed reproductive failure.

**Conclusions:**

DA crosses the placenta, enters brain tissue of prenates, and accumulates in the amniotic fluid. Amniotic fluid appears to be a useful fluid to monitor DA exposure.

The marine toxin domoic acid (DA), produced by the cosmopolitan diatom species *Pseudonitzchia*, affects numerous organisms in the wild through trophic transfer, including sea birds, manatees, dolphins, and sea lions, as well as humans (Gulland et al. 2002; Lefebvre et al. 1999; Perl et al. 1990; Scholin et al. 2000; Work et al. 1993). DA is an excitotoxin that binds to kainate subtypes of ionotropic glutamate receptors as a high affinity partial agonist that prevents normal channel inactivation, leading to depolarization and release of glutamate. This then activates NMDA (*N*-methyl-d-aspartate) ionotropic receptors to excite seizure-prone circuitry in the limbic region of the brain (Ramsdell 2007). The behavioral effects of DA exposure, such as scratching, ataxia, tremors, and seizures, are well documented in various adult experimental organisms including mice, rats, and cynomolgus monkeys (Iverson et al. 1989; Tasker et al. 1991; Tryphonas et al. 1990a, 1990b). These same symptoms have been documented in stranded California sea lions exposed to DA (Gulland et al. 2002). Prolonged neuronal excitation caused by exposure to DA can also cause damage to the hippocampus (Peng et al. 1994), which has been linked to learning (Clayton et al. 1999) and memory deficits in experimental animals as well as humans and marine mammals (Brodie et al. 2006; Petrie et al. 1992; Scholin et al. 2000; Sutherland et al. 1990; Teitelbaum et al. 1990).

An increasing number of studies have examined the developmental effects of prenatal DA exposure, although it is still relatively poorly understood. Already researchers have determined that exposure to nonsymptomatic levels of DA *in utero* can cause neurologic and behavioral effects that persist even into adulthood. When mice were exposed prenatally, severe hippocampal degradation was observed 14 days after birth (Dakshinamurti et al. 1993). Neonatal rats were shown to be more susceptible to DA exposure than adults, most likely caused by insufficient clearance of DA due to immature renal function (Doucette et al. 2000; Xi et al. 1997). Memory loss and learning deficits persist in embryonic rats exposed to DA in midgestation (Levin et al. 2005), but animals are less affected when exposed in late gestation (Levin et al. 2006). These previous studies clearly demonstrate that nonsymptomatic doses of DA can cause neurologic and developmental damage when organisms are exposed prenatally. Studies have also addressed how the toxin crosses from plasma to brain tissue through the blood–brain barrier (BBB), presumably through the capillary endothelium. Johansson et al. (2006) used tracers similar in size and permeability to DA and determined that the tight junctions on capillary endothelial cells, generally assumed to be a major factor for the impermeability of small polar molecules into the brain, are in place and operating very early in development.

The importance of understanding how DA partitions between the exposed mother and the developing fetuses during an *in utero* exposure experiment can help better determine how DA affects naturally exposed humans and animals and the reproductive health impacts thereof. No confirmed cases of DA intoxication in humans have been reported since the 1987 human exposure in Canada (Perl et al. 1990); however, subsequent identification of DA in shellfish in the Pacific Northwest has lead to concern of potential human intoxications, especially in communities that are subsistent on local shellfish resources. DA intoxication has been shown to be responsible for 9% of all sea lion strandings on the West Coast (Greig et al. 2005) and is one cause of reproductive failure seen in California sea lions. In 1998 and 2002, 209 combined strandings of pregnant California sea lions were attributed to DA toxicity, with transfer of DA from the mothers to the young *in utero*; all 209 animals subsequently showed reproductive failure in the form of spontaneous abortion, premature birth, or the mortality of the female (Brodie et al. 2006). These observances indicate the necessity of good biomonitoring capabilities, as well as the need to understand the mechanisms and impacts of toxins on marine mammal populations, especially when reproductive processes are susceptible.

To better determine how much DA is transferred from an exposed mother to its young *in utero*, we measured DA concentrations in maternal plasma and brain tissue, amniotic fluid, and fetal brain tissue. We anticipate using this information to understand the dose response to DA exposure, the partitioning of DA in tissues, and the usefulness of amniotic fluid as a marker of fetal exposure. Identification of physiologically relevant concentrations of DA in amniotic fluid could be used to score potential toxicity effects later in life, as well as to understand the magnitude to which it may contribute to the observed premature parturition seen in the West Coast. This will also aid in treatment of those animals that are determined to have been exposed.

## Methods

### Rat exposure

All animal exposures were performed by Argus Research (Horsham, PA), a division of Charles River Laboratories, Inc. (Boston, MA). Experiments were carried out according to the *Guide for the Care and Use of Laboratory Animals* (Institute of Laboratory Animal Resources 1996), and animals were treated humanely and with regard to alleviation of suffering. Thirty-four female rats (281–387 g) and their respective litters were provided by Charles River Laboratories, Inc., and exposed dams were divided into six treatment groups (Table 1). Our sublethal doses were chosen based on previously used doses that showed hippocampal damage during fetal development (Dakshinamurti et al. 1993; Levin et al. 2005); initial range-finding exposures showed that 0.6 mg/kg body weight (bw) was nonsymptomatic to the dam, and 1.6 mg/kg caused minimal observational effects. Groups I, II, and III were injected intravenously (iv) on gestational day (GD) 13 and groups IV–VI were injected (iv) on GD20. Groups II and V were administered a nonlethal dose of 0.6 mg/kg (0.1 mg/mL) DA (Sigma-Aldrich, St. Louis, MO) in phosphate-buffered saline (PBS); groups III and VI were injected (iv) with 1.6 mg/kg DA; and groups I and IV were injected with an equal volume of control vehicle (PBS) only.

All rats were sacrificed at 1 hr post-exposure. Blood was collected from dams, transferred into dipotassium EDTA-coated tubes, and centrifuged to collect the plasma fraction, which was then frozen (< ^−^20°C) until shipment for analysis. Amniotic fluid samples were also collected from each dam, pooled by litter, and frozen (< ^−^20°C) until shipment for analysis. Fetuses were removed from the uterus, and whole brain tissues were removed, individually snap frozen in liquid nitrogen, and maintained frozen (< ^−^68°C) until shipment for analysis. Maternal rat brains were removed, and three brain regions were dissected out (frontal cortex, hippocampus, and hypothalamus) from this experiment at 1 hr postexposure. In a second experiment, the same maternal brain sections were collected from rats dosed at 1.6 mg/kg bw 2–4 hr after exposure; however, the brains were vascularly perfused with saline before dissection.

### Tissue extraction

While still frozen, each brain or brain section was weighed, minced, and suspended in an equal volume of 10 mM PBS with 10% methanol and 0.05% Tween 20 (sample/standard buffer). Tissues were extracted by the addition of three times homogenate volume of 50% methanol and homogenized with a hand-held Teflon homogenizer in a 0.05-mL microcentrifuge tube (Kimble-Kontes, Vineland, NJ). Extracts were centrifuged at 3,000 × *g* (IEC Centra CL2 Benchtop centrifuge; Thermo Electron Corporation, Waltham, MA) and the supernatant removed for further analysis. All samples and sample extracts were diluted in sample/standard buffer, with plasma diluted 5,000- to 10,000-fold, amniotic fluid diluted 500-fold, and brain tissue diluted 40- or 100-fold in order to fall in the working range of the assay.

### ASP direct cELISA kit

ASP (amnesic shellfish poison) direct cELISA kits from Biosense Laboratories (Bergen, Norway) were used for the analysis of all plasma, amniotic fluid, and brain tissue samples. This kit uses a polyclonal ovine anti-DA antibody conjugated to horseradish peroxidase (HRP), to which free DA in samples or standards compete with DA-conjugated proteins coated on the plate well surface (Garthwaite et al. 1998). Samples were incubated with the antibodies, washed with PBS with 0.05% Tween 20, and then treated with TMB (3,3′,5,5′-tetramethyl-benzidine), which reacts with the HRP enzyme to form a blue end product. Addition of 0.3 M sulfuric acid turned the blue to yellow, and the plate was read on a NovoStar plate reader (BMG Labtechnologies, Durham, NC) at 450 nm. Analyses of DA concentrations in the standard curve and samples were performed using ELISA data processing software provided by Biosense Laboratories. Additional analyses were done using Prism 4.0 (GraphPad, San Diego, CA).

## Results

### GD13 animals

**Figure 1:**
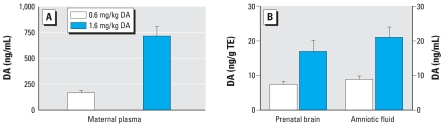
Toxin distribution in tissues of rats exposed to DA on GD13. (*A*) Concentration of DA in maternal blood plasma (*n* = 5). (*B*) Concentration of DA in fetal brain tissue and amniotic fluid (*n* = 10). Error bars indicate SE. All controls were below the limit of detection.

The DA-exposed dams had high plasma levels of DA, with the low-dose group (0.6 mg/kg) having an average of 166 ng DA/mL and the high-dose group (1.6 mg/kg) an average of 716 ng/mL 1 hr postexposure (Figure 1A). The limit of quantification (LOQ) for plasma samples was < 1 ng/mL. All prenates at both doses had detectable levels of DA in their brain tissue and amniotic fluid. Brain tissues from the 0.6-mg/kg group (*n* = 5) averaged 7.5 ng DA/g tissue equivalents (TE), and the 1.6 mg/kg group (*n* = 10) had an average of 16.9 ng DA/g TE (Figure 1B). No matrix effects were seen in the 1:40 dilution of brain tissue. The LOQ for brain tissue at the 1:40 dilution was 0.4 ng/mL, or 2.8 ng DA/g TE. Amniotic fluid concentrations averaged 8.7 and 21.0 ng/mL for the low- and high-dose groups, respectively (Figure 1B). The LOQ for amniotic fluid was < 1 ng/mL.

### GD20 animals

**Figure 2:**
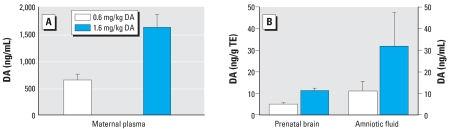
Toxin distribution in tissues of rats exposed to DA on GD20. (*A*) Concentration of DA in maternal blood plasma (*n* = 5). (*B*) Concentration of DA in fetal brain tissue and amniotic fluid (*n* = 10). Error bars indicate SE. All controls were below the limit of detection.

The plasma levels of DA in the exposed mothers at the later gestational date had the highest concentrations, with the low-dose group having an average of 654.7 ng DA/mL and the high-dose group an average of 1,600 ng/mL 1 hr postexposure (Figure 2A). Again, all prenates at both doses had detectable levels of DA in their brain tissue; however, the levels were similar to those in the GD13 group. Prenate brain tissues in the 0.6-mg/kg group (*n* = 10) averaged 5.3 ng DA/g TE; the 1.6-mg/kg group (*n* = 10) had an average of 11.4 ng DA/g TE (Figure 2B). Amniotic fluid concentrations averaged 11.1 and 31.2 ng/mL for the low- and high-dose groups, respectively (Figure 2B). The LOQ values were the same as for the GD13 animals (noted above).

### Maternal brain DA concentrations

The brains of the dams in the 1.6-mg/kg group contained averages of 18, 29, and 24 ng DA/g TE for the frontal cortex, hypothalamus, and hippocampus, respectively. The concentration of DA in brain tissue was not reduced by rinsing blood from the vasculature by saline perfusion (Table 2). The brains of dams in the low-dose group showed lower concentrations of toxin (5, 5.5, and 4.3 ng DA/g TE for the frontal cortex, hypothalamus, and hippocampus, respectively).

## Discussion

The concern regarding the transfer of toxins or other harmful substances from mother to young and the possible *in utero* effects extends beyond humans alone, especially when marine mammals are known to be susceptible to DA intoxication events with ramifications for their offspring. In the present study, using rats as a model, we have shown that DA not only crosses the placenta but also can reach the brain tissue of prenates and accumulate in the amniotic fluid. Here we focus on these concentrations of DA in the context of being toxicologically relevant to fetal development not only to our experimental animals but also potentially to animals naturally exposed to DA, such as the California sea lion.

The DA doses used in this study were both nonsymptomatic to the pregnant rats; however, previous studies using similar doses have been shown to cause neurologic and behavioral effects later in life for the prenates. Prenatal mice exposed *in utero* to 0.6 mg/kg DA on GD13 showed no pathological effects at birth; however, at 14 days of age, hippocampal damage was evident with a decreased seizure threshold to subsequent DA exposure (Dakshinamurti et al. 1993). Levin et al. (2005) found several latent neurobehavioral effects at similar DA doses (0.6 and 1.2 mg/kg) given to rats on GD13. Prenatal rats exposed at this developmental time point had no observable toxicity effects, but they showed decreased locomotor activity, a diminished sex-ratio differential in spatial discrimination learning, and a dose-dependent increase in the amnesic response to scopolamine when tested later in adolescent and early adult life (Levin et al. 2005). When prenatal rats were exposed on postnatal days 1 and 2 (total gestation is 21 days) and underwent the same testing regime as the GD13 rats, Levin et al. (2006) found fewer impairments to neurobehavioral function, with hypoactivity being the only significant effect observed. This suggests that rats are more sensitive to delayed DA effects with exposure on GD13 and that DA doses which are non-symptomatic to the mother are sufficient to cause developmental effects in their young when exposed at a susceptible gestational time point such as GD13.

The comparison of DA partitioning in the maternal and prenatal brain tissues showed that the concentrations measured are dose dependent and relatively similar to each other. This suggests that the brains of the prenates are exposed to roughly the same DA dose as their mothers despite the protective barrier of the placenta and/or the BBB. DA initially enters the brain of adults, presumably at the level of capillaries, with a transfer constant of 12 μg/kg for a low-level exposure to radiotracer DA (Preston and Hynie 1991). Kim et al. (1998) modeled partitioning of DA in various brain regions of the rat using the data of Preston and Hynie (1991) and predicted that 70 pg/g TE would accumulate after 1 hr in the frontal cortex at the DA radiolabel tracer concentration of 7 μg/kg. This converts to 16 ng/g TE for a 1.6-mg/kg dose used in the present study. This theoretical value is very close to our measured value of 18 ng/g TE in the maternal frontal cortex. Because the maternal blood concentration of DA was very high at this dose and time, we were concerned that blood contamination elevated our values, but our comparison to perfused brain sections did not reduce the toxin level in the brain.

The concentration of DA in the brain of the prenates was approximately half of the average maternal brain concentration at GD20, and roughly equal at GD13. This indicates that upon *in utero* exposure, the prenates and mothers are exposed to similar brain DA concentrations at a level previously shown to activate ionotropic glutamate receptors. Xi and Ramsdell (1996) showed that 16 ng/ml DA is sufficient to induce calcium entry into hippocampal pyramidal cells in adults, and it is evident from other studies (Dakshinamurti et al. 1993; Levin et al. 2005) that even the low-dose exposure of 0.6 mg/kg on GD13 is sufficient to have an adverse effect on neurodevelopment. The effective brain concentrations for DA are likely higher (approximately 7-fold) than the TEs would suggest because of the low extracellular water space in the brain. Hence, the brain DA concentrations are likely to be several-fold higher than threshold values in cultures of neurons.

We found the tissue concentrations of DA in the prenatal brain to be similar to the concentrations of DA in the amniotic fluid. Our findings parallel those of Johansson et al. (2006) in their investigation of the permeability of a small, water-soluble marker similar in size to DA (^14^C-sucrose; molecular weight of DA is 311) in GD13 and GD18 prenates. Their results showed the cerebrospinal fluid (CSF)/plasma marker concentration ratios to be 100% on GD13, with a decrease to 39% on GD20 (Johansson et al. 2006). Although early studies indicated that tight junctions between the brain and endothelial cells of the blood capillaries form the functional BBB in adults (Brightman and Reese 1969; Reese and Karnovsky 1967), subsequent studies have indicated an alternative mechanism of entry of small polar molecules into the brain of prenatal animals (Johansson et al. 2006). Tight junctions of brain–blood capillaries are fully functional at least as early as GD15, whereas the barrier between the brain and CSF develops later. With consideration to these studies, our results indicate that the DA enters the GD13 brain without restriction and, even though capillary tight junctions are fully functional shortly afterward, DA continues to enter the brain later in prenatal development with approximately a 50% restriction.

DA accumulates within 1 hr in the amniotic fluid in GD13 and GD20 rats, indicating that it readily crosses the placental barrier and is excreted by the prenates. The DA levels we measured in this experiment (8–21 ng/mL) fell within the same range of values (4–34 ng/mL) measured by Brodie et al. (2006) in stranded pregnant sea lions. The amniotic fluid and brain DA levels may be in equilibrium earlier during *in utero* development, because fetal skin is highly permeable to polar substances as it is not keratinized until later in gestation. Additionally, the primary dermal barrier, which results from the deposition of lipids into the zona corneum, does not become functional in rats until the glucocorticoid surge just before birth (Aszterbaum et al. 1993). Hence, DA in amniotic fluid likely penetrates the skin of earlier-gestation prenates readily.

Although elimination rates for DA from amniotic fluid remain to be conducted, analysis of other polar compounds with similar plasma elimination rates suggest that DA will not eliminate readily from the amniotic fluid (Alnouti et al. 2004). Poor elimination of DA from amniotic fluid is also consistent with the finding of DA in the amniotic fluid of California sea lions. DA found in the amniotic fluid of one animal was detected 8 days after the mother stranded (Brodie et al. 2006); this concentration is comparable to our experimental rat values after only a 1-hr exposure. This suggests that the recirculation of amniotic fluid acts as a sink for DA and continues to expose the fetus long after the DA has been cleared by the mother. The sea lion fetus also had comparable DA levels in gastric fluid and amniotic fluid, which suggests that these animals also had the potential of *in utero* oral exposure to DA (Brodie et al. 2006).

The GD13 developmental stage seems to be a point of vulnerability for developmental toxicity to DA; however, the potential of continued absorption of DA from the amniotic fluid by dermal exposure may place the timing of DA action on neurodevelopment somewhat later. GD13 is a critical developmental point in the mouse, as it is the beginning of neurogenesis of the hippocampus (Angevine 1965; Haubensak et al. 2004), and the rat reaches this point a day or two later (Bayer 1980). Exposure during this stage might interfere with the clonal expansion and/or migration of neuroprogenitor cells. A toxic effect of DA on regional brain development is supported by histochemical findings that the hippocampus does not form properly later in postnatal life of GD13 prenates exposed to DA (Dakshinamurti et al. 1993). GD20 approaches the end of neuroproliferation and migration of cells in the amnion horn of the hippocampus, and fewer behavioral effects are observed in animals exposed to DA the first 2 days after birth (Levin et al. 2006). Hence, sustained *in utero* exposure via amniotic fluid suggests that the window for DA toxicity for brain development still needs to be narrowed between GD13 and GD20.

The present study is the first experimental determination of DA in biological fluids and brain tissue of pregnant rats and their fetuses. Amniotic fluid appears to be a promising indicator of previous DA exposure in stranded pregnant sea lions, especially because it seems to be retained at measurable DA levels even after DA has cleared from maternal plasma. Our study showed similar measurable amounts to those found in the wild. We report toxicologically relevant levels of DA in the brain of developing fetuses, although the exact transport mechanisms and the kinetics of DA transport across the either the BBB or CSF–blood barrier are still undetermined. In light of the results from environmental exposures of California sea lions and associated prenatal deaths, identification of *in utero* effect levels for DA toxicity will be beneficial to future health assessment.

## Figures and Tables

**Table 1:** Designation of groups based on DA exposure dose and gestational time point.

	GD13			GD20		
	(*n* = 10)			(*n* = 10)		
Dose (mg/kg bw)	0	0.6^a^	1.6	0	0.6	1.6
Group	I	II	III	IV	V	VI

a *n* = 5

**Table 2:** Comparison of DA (mean ng DA/g TE) in maternal brain sections from pregnant rats exposed to DA at 1.6 mg/kg bw.

Brain section	Perfused (*n* = 4)	Nonperfused (*n* = 2)
Frontal cortex	20.26	18.05
Hypothalmus	44.01	29.31
Hippocampus	31.69	24.20
